# Light-curing units used in dentistry: factors associated with heat development—potential risk for patients

**DOI:** 10.1007/s00784-016-1962-5

**Published:** 2016-10-01

**Authors:** Mathieu Mouhat, James Mercer, Lina Stangvaltaite, Ulf Örtengren

**Affiliations:** 1 Department for Clinical Dentistry/Faculty of Health Sciences, The Arctic University of Norway (UIT), Tromsø, Norway; 2 Department of Medical Biology/Faculty of Health Sciences, The Arctic University of Norway (UIT), Tromsø, Norway; 3 Department of Cariology, Institute of Odontology/Sahlgrenska Academy, University of Gothenburg, Gothenburg, Sweden

**Keywords:** Dentistry, Curing lights, Tooth, Light, Temperature

## Abstract

**Objectives:**

To investigate how heat development in the pulp chamber and coronal surface of natural teeth with and without cusps subjected to irradiance using light-emitting diode (LED)–light-curing units (LCUs) is associated with (i) irradiance, (ii) time, (iii) distance, and (iv) radiant exposure.

**Materials and methods:**

Three different LED-LCUs were used. Their irradiance was measured with a calibrated spectrometer (BlueLight Analytics Inc., Halifax, Canada). An experimental rig was constructed to control the thermal environment of the teeth. The LED-LCU tip position was accurately controlled by a gantry system. Tooth surface temperature was measured by thermography (ThermaCAM S65 HS, FLIR Systems, Wilsonville, USA) and pulp chamber temperature with a thermocouple. LED-LCU tip distance and irradiation times tested were 0, 2, and 4 mm and 10, 20, and 30 s, respectively. Ethical permission was not required for the use of extracted teeth.

**Results:**

Maximum surface and pulp chamber temperatures were recorded in tooth without cusps (58.1 °C  ± 0.9 °C and 43.1 °C ± 0.9 °C, respectively). Radiant exposure explained the largest amount of variance in temperature, being more affected by time than irradiance.

**Conclusions:**

At all combinations of variables tested, repeated measurements produced consistent results indicating the reliability of the method used. Increased exposure time seems to be the factor most likely to cause tissue damage.

**Clinical relevance:**

Risk of superficial tissue damage at irradiances >1200 mW/cm^2^ is evident. There is a risk of pulp damage when only thin dentin is left at higher irradiances (>1200 mW/cm^2^). Clinicians should be aware of LED-LCU settings and possible high temperature generated.

## Introduction

Polymer resin-based materials (e.g., adhesives, composites, and composite cements) are widely used in restorative dentistry. Photopolymerization dominates using blue light of a wavelength between 380 and 500 nm and with an irradiance of >450 mW/cm^2^. During the last 10 years, light-curing units (LCUs) employing light-emitting diode (LED) technology have largely taken over from the older quartz-tungsten-halogen (QTH) devices [1–3]. LED is considered as “cold light” created by recombination of electrons using crystals (e.g., GaN) releasing photons (i.e., light) when subjected to energy (i.e., voltage) [4]. The advantage of LED is a spectrum closer to the point for photoexcitation of the most widely used photoinitiators in dental resin-based composites (RBCs). In addition, use of energy is more efficient (i.e., more light and less heat) [3].

In recent years, light-curing unit employing light-emitting diode (LED-LCU) with higher energy output than previous generations of LCU have emerged on the market. The reason for this development trend is claimed to be shorter curing times and increased polymerization, although the latter is still a matter for discussion and has been questioned [5, 6]. With higher output, there is a risk of increased temperature even with LED technology and concerns have been raised about increasing risk for pulp and tissue damage in patients [1, 2, 7, 8], a risk which also was of concern with QTH devices [9–11]. Hanning and Bott investigated QTH curing devices and showed a temperature increased of ≈8 °C depending on energy output [11]. If the temperature of the pulpal tissue increases more than 5.5 °C, the tissue will start to coagulate, causing irreversible damage [12].

Although few investigations have been performed, especially with LED-LCU, an increase in energy output (irradiance) seemed to correlate best to increased temperature [5]. Complaints from patients in connection with light-curing procedures have been reported, including experience of “burning” sensations in teeth and in oral tissue [13].

Different brands of LED-LCU with the same expected output do not always produce the same amount of heat, and this may be due to differences between the light in spectral distribution [14], the type of LED-LCU tip (TIP) used and its diameter, and/or the use of a fan in the LED-LCU. In addition to the heat produced by the LED-LCU, the polymerization of the composite (i.e., exothermic reaction) has been discussed as causes for tissue damage (i.e., pulp damage) [8, 11, 15, 16]. The light source is still, however, considered to be the main risk [17], even though the composite and the remaining dental hard tissue may give some protection [18].

There are major concerns from the authorities (e.g., EU) concerning risk of tissue damage in patients. Different methods have been used to shed more light on this problem. However, the results obtained vary with large discrepancy between different LED-LCUs [19–22]. The majority of the studies have looked into the matter of pulp damage, but there is also a risk for other tissue damage close to the TIP. By investigating the association of the irradiance with the temperature increase close to the TIP and in the pulp chamber using different types of LED-LCU at increased distances and curing times, more knowledge can be added for decreased risk for patients. While the temperature distribution on RBC has been investigated [23], to the best of our knowledge, there are no studies simultaneously measuring and combining the temperature distribution on the surface and in the pulp chamber in teeth subjected to LED-LCU.

The aim of this study was to develop a reliable bench model for investigating how heat development in the pulp chamber and coronal surface of natural teeth with and without cusps subjected to irradiance using three different LED-LCU is associated with (i) irradiance, (ii) time, (iii) distance, and (iv) radiant exposure (product of irradiance and time which represent the total light energy delivered to the RBC).

## Materials and methods

### LED-LCU tested

Two different brands of LED-LCU were tested, Bluephase style® and Bluephase G2®. One LED-LCU Bluephase G2® was tested in two modes (high mode and low mode). Two LED-LCU Bluephase style® were tested, one battery powered and one mains powered. These units are in everyday use in our university dental clinic, and complaints have been raised from patients suffering from pain caused by heat development during light curing.

### Irradiance measurement

Two different brands of LED-LCU, Bluephase style® (*n* = 10) and Bluephase G2® (*n* = 10), from the same manufacturer (IvoClar/Vivadent, Schaan, Lichtenstein) were tested for irradiance using a calibrated laboratory-grade NIST-referenced USB4000 spectrometer (Managing Accurate Resin Curing (MARC) System; Bluelight Analytics Inc., Halifax, Canada). The objective was to evaluate eventual differences in irradiance among the curing unit within the same brand. The LED-LCU were battery powered, the batteries being fully charged on all test occasions. The working capacity of the LED-LCU lithium-polymer battery for the two brands of LED-LCU is ≈60 min. Bluephase G2® was tested in high mode. One Bluephase style® was also tested when connected to main electricity. Caution was taken in the precise placement of the TIP on the sensor of the measurement equipment. To achieve this, we used an adjustable precision gantry with a 0.1-mm scale (#55025, Edmunds Optics, Barrington, NJ). For evaluation of differences among units in the same brand, five measurements for each unit at 0-mm distance were conducted at irradiation times of 10, 20, and 30 s, respectively. The variation in irradiance was small for 9 out of 10 units in the same brand. One of these 9 units for each brand was randomly selected for the temperature profile experiments.

### Preparation of teeth

For the temperature profile experiments, caries-free human third molars were used. The teeth were extracted for surgical reasons and not older than 6 months. They were stored in 0.5 % chloramine-T solution according to ISO/TS 11405–2015 in a refrigerator (4 ± 1 °C) prior to use. In one tooth (T1), a class I cavity was prepared with a cylindrical diamond (Ø = 1.2 mm) bur through the enamel and into the dentin. The apex of the root was cut and the channel prepared up to the pulp chamber with K-files (Densply/Maillefer, Ballaigues, Switzerland) 35 and 70. The tip of a thin (0.2 mm) type T (copper constantan) thermocouple was inserted into the pulp chamber via the prepared channel, and its position was controlled with radiography (Planmeca Intra X-ray unit with Romexis, Planmeca Oy, Helsinki, Finland). The remaining pulpal wall had a thickness of 1.3 ± 0.2 mm as assessed from the radiograph. In order to avoid an air space surrounding the tip of thermocouple inside the pulp chamber, the prepared channel was filled with glycerol prior to insertion of the thermocouple. The excess glycerol (spillage) during insertion was removed. Glass Ionomer (Fuji I®, GC Corp., Tokyo, Japan) was used to seal the apex and secure the thermocouple.

Due to the unevenness of the occlusal surface, the distance from the TIP to the pulpal wall is quite large. Under certain clinical conditions, for example, a tooth with removed cusps, the TIP will come closer to the pulpal wall. This situation was also taken into account in the experimental design and involved a slightly different preparation of the tooth under investigation. A second tooth (T2) was cut in the horizontal plane using a diamond saw (Accutom 50, Struers, Ballerup, Denmark), creating a flat dentin surface with a pulpal wall thickness of approximately 0.6 mm. Radiography was also used to control placement of the thermocouple and thickness of the dentin wall as described above.

### Controlling the baseline thermal environment

**Fig. 1:**
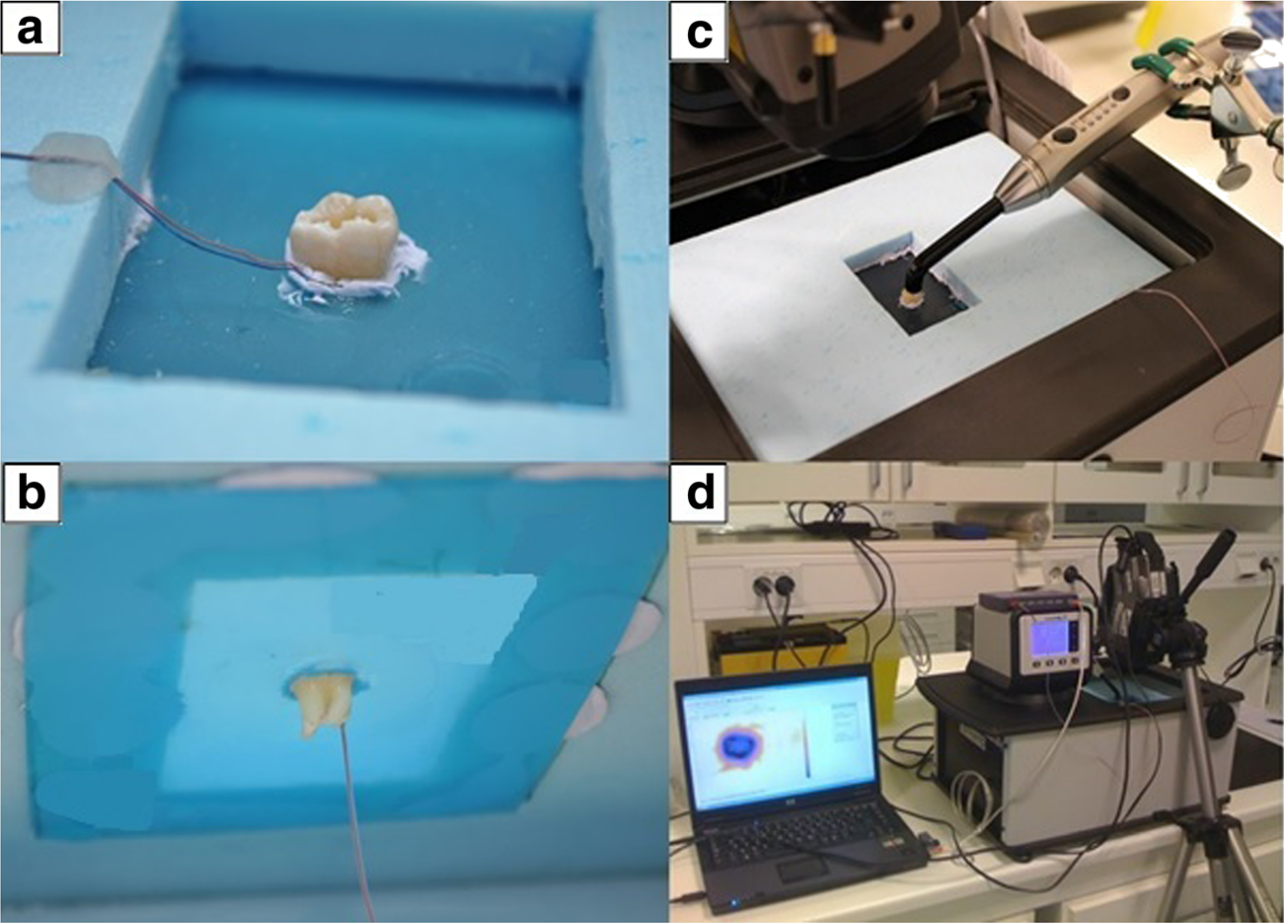
**a**, **b** The placement of a tooth in the thin plastic sheet with the root visible on one side and the crown on the other are shown. During an experiment, the thin plastic sheet with the mounted tooth was placed on the water surface, such that the root was submerged in the water. **c** The position of the LED-LCU being tested. Overview of the experimental setup (**d**) showing the position of the infrared camera over the opening of the water bath during an experiment

In an attempt to simulate as closely as possible the thermal conditions within the oral cavity, a special experimental rig was constructed (Fig. 1), involving the use of a thermostatically controlled circulating water bath (AH15L HT, VWR International, Radnor, USA) maintained at 37 ± 1 °C. The individual tooth under investigation was inserted approximately halfway (at the cemento-enamel junction) through an opening in the centre of a 75 × 50 × 1.25-mm-thick plastic sheet, in such a way that the root was visible on one side and the crown on the other. The edges of the plastic plate were in turn glued to one side of a 12-mm-thick sheet of expanded polystyrene that had a rectangular-shaped opening with slightly smaller dimensions to the plastic plate. The plastic plate was attached to the polystyrene plate with the coronal side of the tooth situated within the open space of the polystyrene plate. This combined unit was then positioned on the water surface inside the thermostatically controlled circulating water bath, such that the root was immersed below the water surface and the coronal part in the air (Fig. 1b). A second thermocouple was placed in the air space ≈2 mm from the coronal side of the tooth for measuring the air temperature in the immediate vicinity of the tooth. The thermocouples used were calibrated prior to the experiment against a certified reference thermometer than had been calibrated against a traceable reference source (Norwegian Standards Organization). The temperature accuracy was 0.1 °C.

The surface temperature of the tooth was measured by thermography using a high-precision infrared camera (ThermaCAM S65 HS, FLIR Systems, Wilsonville, USA) with a close-up lens (LW64/150). This camera can produce high-definition digital IR images with an accuracy of 0.1 °C. The thermographic temperature data were recorded at 1-s intervals and stored for later analysis. The thermal emissivity was set at 0.98. The thermographic images (thermograms) were processed using ThermaCAM Researcher Pro 2.8 SR-2 (FLIR Systems, Wilsonville, USA). The infrared camera was regularly calibrated against a black body with a traceable temperature source (model IR-2103/301, Infrared Systems Development Corporation, FL, USA). Although the absolute accuracy of this infrared camera is 30 mK, the processed temperature data was rounded off to 0.1 °C.

For temperature measurements, the LED-LCU chosen from the irradiance test (Bluephase style® #1100015231, Bluephase G2® #P626170S591130, and Bluephase style® electrically powered unit #1100008001) were tested at the following combinations of curing time and distance from the tooth surface (10, 20, and 30 s and 0, 2, and 4 mm, respectively). The curing times chosen are within the range recommended by the manufacturers. The chosen distances of the TIP from the tooth were based on those generally used in a clinical setting. For Bluephase G2®, the tests were performed both in low (≈700 mW/cm^2^) and high modes (≈1400 mW/cm^2^). Five repeated measurements for each distance/time combination were performed. All temperature data were continuously recorded before, during, and after a simulated curing cycle. Between each measurement, a recovery time was allowed to make sure that the temperatures had returned to its baseline value (pre-irradiation value).

## Ethical permission

Since the experiments involved the use of human material (i.e., extracted teeth), ethical permission was asked for from the Norwegian Regional Committee for Medical and Health Research Ethics (REK). The committee concluded that such permission was not required (2015/234/REK Nord).

## Statistics

In the analysis of the experimental data, the null hypothesis formulated was that the heat development is not associated with (i) irradiance, (ii) time, and (iii) distance. Statistical analyses were carried out using Statistical Package for the Social Sciences (SPSS, version 22.0, IBM, Somers, NY, USA). Statistical evaluation was performed using Wilcoxon rank sum test for comparisons between means of irradiance. Eight multiple linear regression models were constructed to evaluate heat development on the surface and the pulp chamber (dependent variables) on T1 and T2. In addition, 16 multiple regression models were constructed in order to compare the influence on temperature of different LED-LCU and curing modes (Bluephase style® electrically powered, Bluephase style® battery, Bluephase G2® high mode, and Bluephase G2® low mode) on T2. The first type of models (model 1) included irradiance, time, and distance as independent variables, while the other type models (model 2) included radiant exposure (product of irradiance and time) and distance. Forced entry method was used. *R* ^2^ was recorded for the whole model and for each variable in order to evaluate their impact on the variation in the temperature. The level of significance was set to 5 % and confidence interval to 95 %.

## Results

### Irradiance measurement

The mean values of irradiance of ten Bluephase style® LED-LCU and mean value of ten Bluephase G2® LED-LCU were statistically significantly different (Table 1). There was also a significant difference in irradiance with time within the ten Bluephase style® units (Table 1).

**Table 1:** Mean (SD) for the irradiance of the two different light-curing units tested, Bluephase style® battery (*n* = 10) and Bluephase G2® high mode (*n* = 10), at three different curing times

Curing time	Irradiance of Bluephase style® battery in mW/cm^2^	Irradiance of Bluephase G2® high mode in mW/cm^2^
10 s	1337 (104) ade	1411 (142) a
20 s	1477 (240) bd	1362 (121) b
30 s	1479 (96) ce	1382 (102) c

The letters a-c indicate significant difference (*p* < 0.05) between Bluephase G2® and Bluephase style@ at the different times tested respectively. The letter d-e indicates significant difference (*p* < 0.05) in irradiance at different times for Bluephase style®. Wavelength for the two light-curing units was 385–515 nm

The irradiance was higher than claimed from the manufacturer for the majority of the units tested (Table 1). For Bluephase style®, the claimed maximum irradiance was 1100 ± 10 % mW/cm^2^ and for Bluephase G2® 1200 ± 10 % mW/cm^2^.

### Temperature measurement

**Fig. 2:**
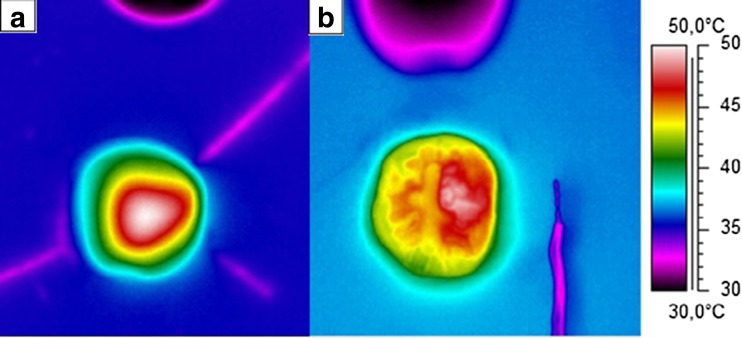
Thermograms showing temperature distribution on the surface of a tooth when subjected to a 30-s irradiation with a LED light-curing unit Bluephase style®. **a** Thermogram of tooth with a flat dentin surface with a pulpal wall thickness of approximately 0.6 mm (T2). **b** Thermogram of tooth with class I cavity (T1)

The temperature distribution on the surface of the T1 was non-uniform compared to T2 (Fig. 2a, b). The increase in pulp chamber temperature was less for T1 compared to T2 (Tables 2 and 3). The maximal surface temperature was 58.1 ± 0.9 °C (for T2 at 2-mm distance, 30-s curing time), and the maximal pulp chamber temperature was 43.1 ± 0.9 °C (for T2 at 0 mm, 30-s curing time) (Tables 5 and 7).

**Table 2:** Temperature variations at the surface and in the pulp chamber at different radiant exposure (product of irradiance and time (s)). Distance from the surface (top of the cusp from tooth with class I cavity (T1)) light-curing unit tip 0 mm. The table shows the results for T1

Light-curing unit (irradiance—mW/cm^2^)	Radiant exposure (J/cm^2^)	Surface temperature (°C)	Pulp chamber temperature (°C)	Time (s)
Bluephase style plugged (1184 ± 12)	35.9 ± 0.4	49.3 ± 0.4	37.6 ± 0.1	30
	23.9 ± 0.3	45.6 ± 0.1	37.2 ± 0	20
	12 ± 0.2	41.2 ± 0.2	36.9 ± 0	10
Bluephase G2 high mode (1471 ± 65)	44.1 ± 1.9	53.1 ± 0.3	37.9 ± 0.1	30
	29.4 ± 1.3	48.2 ± 0.4	37.4 ± 0.1	20
	14.6 ± 0.8	43.7 ± 0.3	37.1 ± 0	10
Bluephase G2 low mode (767 ± 46)	23.0 ± 1.4	45.7 ± 0.2	37.3 ± 0	30
	15.3 ± 0.9	42.8 ± 0.6	37.1 ± 0.1	20
	7.7 ± 0.5	39.6 ± 0.3	36.9 ± 0	10
Bluephase style battery (1268 ± 21)	38.1 ± 0.6	49.5 ± 0.3	37.6 ± 0.1	30
	25.4 ± 0.4	46.3 ± 0.3	37.3 ± 0	20
	12.7 ± 0.2	43.1 ± 0.4	37.1 ± 0	10

**Table 3:** Temperature variations at the surface and in the pulp chamber at different radiant exposure (product of irradiance and time (s)). The table shows the results for the tooth with a flat dentin surface with a pulpal wall thickness of approximately 0.6 mm (T2). Distance from the surface to the light curing unit tip 0 mm

Light-curing unit (irradiance—mW/cm^2^)	Radiant exposure (J/cm^2^)	Surface temperature (°C)	Pulp chamber temperature (°C)	Time (s)
Bluephase style plugged (1257 ± 4)	37.8 ± 0.1	44.4 ± 0.8	40.8 ± 0.2	30
	25.1 ± 0.1	42.5 ± 0.4	39.2 ± 0.1	20
	12.6 ± 0.1	40.1 ± 0.3	38.4 ± 0.1	10
Bluephase G2 high mode (1437 ± 14)	43.1 ± 0.4	46.9 ± 1.2	43.1 ± 0.9	30
	28.7 ± 0.3	45.3 ± 1.2	41.4 ± 0.3	20
	14.6 ± 0.1	42.5 ± 0.3	39.1 ± 0.2	10
Bluephase G2 low mode (774 ± 7)	23.2 ± 0.2	41.5 ± 0.3	39.1 ± 0.2	30
	15.5 ± 0.1	40.3 ± 0.3	38.5 ± 0.2	20
	7.7 ± 0.1	38.1 ± 0.2	37.2 ± 0.1	10
Bluephase style battery (1222 ± 9)	36.7 ± 0.3	52.6 ± 0.5	42.5 ± 0.4	30
	24.4 ± 0.2	52.5 ± 0.4	41.5 ± 0.3	20
	12.2 ± 0.1	48.6 ± 0.7	40.0 ± 0.1	10

**Table 4:** Irradiance, radiant exposure (product of irradiance and time (s)), and heat development (surface temperature and pulp chamber temperature) at different distances (on tooth with a flat dentin surface with a pulpal wall thickness of approximately 0.6 mm (T2)) with different light-curing units, Bluephase style® plugged

Distance (mm)	Irradiance (mW/mm^2^)	Time (s)	Radiant exposure (J/mm^2^)	Surface temperature (°C)	Pulp chamber temperature (°C)
0	1257 ± 4	30	37.8 ± 0.1	44.4 ± 0.8	40.8 ± 0.2
		20	25.1 ± 0.1	42.5 ± 0.4	39.2 ± 0.1
		10	12.6 ± 0.1	40.1 ± 0.3	38.4 ± 0.1
2	1250 ± 9	30	37.5 ± 0.3	44.9 ± 0.4	40.5 ± 0.4
		20	25.0 ± 0.2	42.9 ± 0.3	39.6 ± 0.2
		10	12.5 ± 0.1	40.3 ± 0.2	37.9 ± 0.2
4	1176 ± 6	30	35.3 ± 0.2	42.8 ± 0.3	39.4 ± 0.3
		20	23.5 ± 0.1	41.1 ± 0.4	38.3 ± 0.2
		10	11.8 ± 0.1	38.9 ± 0.4	37.2 ± 0.1

**Table 5:** Irradiance, radiant exposure (product of irradiance and time (s)) and heat development (surface temperature and pulp chamber temperature) at different distances (on tooth with a flat dentin surface with a pulpal wall thickness of approximately 0.6 mm (T2)) with different light-curing units, Bluephase G2® high mode

Distance (mm)	Irradiance (mW/mm^2^)	Time (s)	Radiant exposure (J/mm^2^)	Surface temperature (°C)	Pulp chamber temperature (°C)
0	1437 ± 14	30	43.1 ± 0.4	46.9 ± 1.2	43.1 ± 0.9
		20	28.7 ± 0.3	45.3 ± 1.2	41.4 ± 0.3
		10	14.6 ± 0.1	42.5 ± 0.3	39.1 ± 0.2
2	1412 ± 16	30	42.4 ± 0.5	52.2 ± 0.6	42.7 ± 0.6
		20	28.3 ± 0.3	49.1 ± 0.2	41.9 ± 0.3
		10	14.1 ± 0.2	44.6 ± 0.8	39.1 ± 0.2
4	1513 ± 16	30	45.4 ± 0.5	48.7 ± 0.5	40.7 ± 0.8
		20	30.3 ± 0.3	46.2 ± 1.1	39.3 ± 0.3
		10	15.1 ± 0.2	42.8 ± 0.3	37.6 ± 0.1

**Table 6:** Irradiance, radiant exposure (product of irradiance and time (s)), and heat development (surface temperature and pulp chamber temperature) at different distances (on tooth with a flat dentin surface with a pulpal wall thickness of approximately 0.6 mm (T2)) with different light-curing units, Bluephase G2® low mode

Distance (mm)	Irradiance (mW/mm^2^)	Time (s)	Radiant exposure (J/mm^2^)	Surface temperature (°C)	Pulp chamber temperature (°C)
0	774 ± 7	30	23.2 ± 0.2	41.5 ± 0.3	39.1 ± 0.2
		20	15.5 ± 0.1	40.3 ± 0.3	38.5 ± 0.2
		10	7.7 ± 0.1	38.1 ± 0.2	37.2 ± 0.1
2	827 ± 6	30	24.8 ± 0.2	41.1 ± 0.7	39.2 ± 0.3
		20	16.5 ± 0.1	40.5 ± 0.2	38.2 ± 0.2
		10	8.3 ± 0.1	38.2 ± 0.3	37.1 ± 0.2
4	864 ± 6	30	25.9 ± 0.2	42.2 ± 0.3	38.5 ± 0.3
		20	17.3 ± 0.1	40.8 ± 0.3	37.6 ± 0.2
		10	8.6 ± 0.1	38.7 ± 0.3	36.9 ± 0.1

**Table 7:** Irradiance, radiant exposure (product of irradiance and time (s)), and heat development (surface temperature and pulp chamber temperature) at different distances (on tooth with a flat dentin surface with a pulpal wall thickness of approximately 0.6 mm (T2)) with different light-curing units, Bluephase style® battery

Distance (mm)	Irradiance (mW/mm^2^)	Time (s)	Radiant exposure (J/mm^2^)	Surface temperature (°C)	Pulp chamber temperature (°C)
0 mm	1222 ± 9	30	36.7 ± 0.3	52.6 ± 0.5	42.5 ± 0.4
		20	24.4 ± 0.2	52.5 ± 0.4	41.5 ± 0.3
		10	12.2 ± 0.1	48.6 ± 0.7	40.0 ± 0.1
2 mm	1537 ± 13	30	46.2 ± 0.4	58.1 ± 0.9	42.0 ± 0.6
		20	30.7 ± 0.3	51.3 ± 0.6	41.3 ± 0.4
		10	15.4 ± 0.1	47.8 ± 0.8	40.0 ± 0.3
4 mm	1366 ± 1	30	40.1 ± 0.0	53.1 ± 0.8	39.5 ± 0.3
		20	27.3 ± 0.0	50.2 ± 0.4	39.3 ± 0.4
		10	13.7 ± 0.0	46.3 ± 1.3	37.7 ± 0.2

**Table 8:** Influence of curing time (time), distance, irradiance, and radiant exposure (product of irradiance and time) on temperature of the surface and pulp chamber on tooth with class I cavity (T1) and tooth with a flat dentin surface with a pulpal wall thickness of approximately 0.6 mm (T2) according to multiple linear regression analyses

		T1		T2	
		Surface temperature	Pulp chamber temperature	Surface temperature	Pulp chamber temperature
	Independent variable	B (95 % CI) *p* value			
Model 1	Time				
	20 s (vs. 10 s)	4.2 (3.5–4.9) 0.000	0.3 (0.2–0.4) 0.000	2.9 (1.8–4.0) 0.000	1.6 (1.4–1.9) 0.000
	30 s (vs. 10 s)	7.2 (6.5–7.8) 0.000	0.9 (0.8–1.0) 0.000	5.2 (4.0–6.3) 0.000	2.6 (2.3–2.8) 0.000
	Irradiance (per 100 units)	0.8 (0.7–0.9) 0.000	0.001 (0–0.001) 0.000	1.3 (1.1–1.4) 0.000	0.4 (0.3–0.4) 0.000
	Distance				
	2 mm (vs. 0 mm)	−1.5 (−2.2–(−0.9)) 0.000	−0.05 (−0.1–0.4) NS	0.4 (−0.8–1.5) NS	−0.6 (−0.7–(−0.3)) 0.000
	4 mm (vs. 0 mm)	−3.9 (−4.6–(−3.3)) 0.000	−0.3 (−0.4–(−0.2)) 0.000	−0.9 (−2.0–0.3) NS	−1.8 (−2.0–(−1.6)) 0.000
	Explained variance *R* ^2^	0.86	0.84	0.62	0.85
Model 2	Radiant exposure (per 10 units)	2.9 (2.7–3.2) 0.000	0.3 (0.3–0.4) 0.000	3.0 (2.5–3.4) 0.000	1.2 (1.1–1.3) 0.000
	Distance				
	2 mm (vs. 0 mm)	−1.4 (−2.1–(−0.7)) 0.000	−0.03 (−0.1–0.05) NS	0.9 (−0.4–2.3) NS	−0.4 (−0.7–(−0.2)) 0.002
	4 mm (vs. 0 mm)	−3.7 (−4.4–(−3.0)) 0.000	−0.3 (−0.4–(−0.2)) 0.000	−0.5 (−1.8–0.8) NS	−1.7 (−1.9–(−1.5)) 0.000
	Explained variance *R* ^2^	0.84	0.84	0.49	0.81

Multiple linear regression analyses (model 1) showed that time explained most of the variation in the pulp chamber (*R* ^2^ = 0.69) and surface (*R* ^2^ = 0.47) temperature in T1 as well as of pulp chamber temperature in T2 (*R* ^2^ = 0.38) (Table 8).

For T1, increasing the irradiation time from 10 to 20 s caused an increase of surface temperature of 4.2 °C (*p* < 0.001) (Table 8). Extending the irradiation time to 30 s caused the surface temperature to increase by 7.3 °C (*p* < 0.001) (Table 8). There was a statistically significant increase in the surface temperature (0.8 °C) when increasing irradiation by 100 u (mW/cm^2^) (*p* < 0.001) (Table 8). In all experiments, in T1 pulp, chamber temperature was never greater than 37.9 °C (Table 2).

For T2, extending the curing time from 10 to 20 s caused the pulp chamber temperature to increase by 1.6 °C (*p* < 0.001), while extending curing time to 30 s, pulp chamber temperature increased by 2.6 °C (*p* < 0.001) (Table 8).

For T2, the temperature increase on the surface was more explained by irradiance than time (*R* ^2^ = 0.41 and *R* ^2^ = 0.18, respectively, in multiple linear regression analysis model 1). There was a statistically significant increase in the surface temperature (1.3 °C) when increasing irradiation by 100 u (mW/cm^2^) (*p* < 0.001) (Table 8).

For both T1 and T2, radiant exposure was shown to be the most important factor for heat development according to multiple regression analysis model 2. For T1, 69 % of the variation of the surface temperature and 75 % of the pulp chamber temperature were explained by radiant exposure. For T2, the outcomes were 47 % (surface temperature) and 62.5 % (pulp temperature), respectively (Table 8). Since radiant exposure is calculated as a function of *watt × time*/*area*, time will still be the most important factor on the temperature variation.

When the different LED-LCU and curing modes were compared (Bluephase style® electrically powered, Bluephase style® battery, Bluephase G2® high mode, and Bluephase G2® low mode), multiple linear regression analyses similarly showed that time was the most important factor for the temperature variations on T2 (data not shown). Independent variables in these analyses were time and distance, as irradiance had to be removed from the model due to multicollinearity. There was one exception (Bluephase style® battery), where pulp chamber temperature was equally explained by time (*R* ^2^ = 0.46) and distance (*R* ^2^ = 0.47). An increase of the irradiation time caused a pulp chamber temperature increase of 2.5 °C (*p* < 0.001), while increasing the distance from 0 to 4 mm caused a pulp chamber temperature decrease of 2.5 °C (*p* < 0.001).

An increase in surface temperature with increasing distance from 0 to 2 mm was seen for all LED-LCU (Tables 4, 5, 6, and 7). For the Bluephase G2®, this was also seen when the distance was increased to 4 mm irrespective of the mode used. Even though the irradiance (and radiant exposure accordingly) was lower at 2-mm distance for the Bluephase G2® high mode, the surface temperature increased (52.2 ± 0.6 °C at 2 mm vs. 46.9 ± 1.2 °C at 0 mm) when irradiated for 30 s (Table 5). For the same unit in low mode and for the Bluephase style® battery tested, the recorded increase in surface temperature followed the increase in irradiance (and radiant exposure accordingly) at different distances (Tables 6 and 7).

## Discussion

In this study, heat development on the surface and in the pulp chamber in extracted teeth subjected to irradiation with different LED-LCU has been investigated. In such a study, accurate temperature measurements as well as accurate control over the baseline thermal environment of the teeth under investigation is of the utmost importance. Great care was taken to ensure that these conditions were achieved. By being able to accurately control the baseline pulp temperature as well as the air temperature surrounding the crown of the tooth using a thermostatically controlled water bath system, we were able to mimic the thermal conditions within the oral cavity (Fig. 2). With regards to the pulp chamber and tooth coronal surface temperature measurements, great care was taken to ensure that the thermocouples and the high-precision infrared camera used for measuring the temperature responses in the pulp chamber and on the surface of the teeth, respectively, were accurately calibrated. The accurate and stable placement of the teeth in the water bath produced results with low standard deviation, and good repeatability at each of the test situations lend further support concerning the reliability of the method used. However, the experimental setup has some limitations in that it can never completely reflect a true clinical situation. Not least, the pulp chamber in the experimental teeth lacks blood perfusion, which might affect heat exchange from the tooth. In addition, while our laboratory setup allowed for exact and stable placement of the TIP during a light-curing cycle, it is unlikely that this would occur in a clinical situation [24]. Further, a laboratory bench model can never simulate the environmental conditions within the oral cavity. An additional consideration that has to be taken into account when comparing temperature distribution from different LED-LCUs relates to differences in the homogeneity of irradiance from the different TIPs [25, 26]. Price at al. evaluated TIPs and found regions in some TIP area that delivered less than 400 mW/cm^2^, while others delivered more than 4500 mW/cm^2^ [23]. It is assumed that it was probably due to differences in TIP design. Due to the focusing properties of the TIPs, irradiance will be affected with increased distance from the surface [27–29]. The observed paradoxical temperature increase for T2 with increased distance for some LED-LCUs might be due to TIP design affecting the irradiance (Tables 4–7). The observed changes in pulp chamber temperatures did not reflect this issue as did the surface temperature. LED-LCU irradiance tested in the present study was higher than stated by the manufacturer. The matter of discrepancy between the output stated by a manufacturer’s and the actual values measured by researchers has been addressed [23].

Irrespective of the type of the LED-LCU used, surface and pulp chamber temperature increased with curing time and irradiance, with increased curing time being clearly the dominant parameter (Table 8). An adequate curing time is necessary in order to obtain an accurate degree of conversion of the RBC [30]. Even modern RBCs are based on methacrylates that require sufficient irradiance and time for proper curing. With inadequate curing time, there is a risk of less cohesion in the network due to less cross-linking and secondary forces. Increasing the irradiance will only, to a limited degree, compensate for a shortened irradiation time [31, 32]. Price et al. (2014) concluded that for RBC, it could be beneficial to increase the curing time of LED-LCU beyond the manufacturer’s recommendations. [33]. However, increased curing time leads to higher temperature and the negative consequence of high temperature during light curing has been a focal point concerning potential pulpal tissue damage [2, 19–21, 24, 34, 35]. This is especially relevant in relation to worn teeth or teeth with larger destructions having a limited amount of dental tissue. In such a situation, light curing is more likely to cause thermally related pulpal tissue damage [36]. The findings of the present study (Tables 2–8) support the latter. For example, the maximal temperature in the pulp chamber in T2 was 43.1 ± 0.9 °C. In cases of thin dentinal walls, close proximity of the TIP, coupled with an irradiance of above 1200 mW/cm^2^, can cause high temperatures with a risk of coagulation of proteins [12, 36]. For this reason, a risk of pulp tissue damage can occur during restorations of class V cavities.

The observation that the temperature distribution on the surface of the T1 was non-uniform compared to T2 is most likely due to the uneven surface topography of T1. The highest surface temperature observed was 58.1 ± 0.9 °C for T2 and 53.1 ± 0.3 for T1 (Tables 2–7). This finding is in line with previous observations [2, 24]. Such high temperatures potentially are a risk for causing thermal damage of the soft tissue.

Within the limitations of this study, it can be concluded that increased curing time seemed to be the factor most likely to cause temperature rise. When the TIP is close to soft tissue, the risk of damage should be seriously taken into account at irradiances >1200 mW/cm^2^. There is also a risk of pulp damage when only thin dentin is left at higher irradiances. Decreased curing time may reduce the risk for soft and pulpal tissue damage but can have a negative effect on the degree of conversion. In addition, several other possibilities have been discussed to reduce overheating when using LED-LCU such as using external cooling from an airflow, polymerization at intermittent intervals, and placing gauze under the rubber dam to reduce heating the soft tissues under the rubber dam [24]. The results of the present study was limited to three LED-LCU (Bluephase style® electrically powered, Bluephase style® battery, Bluephase G2®) including one LCU having two curing modes (Bluephase G2® high mode and Bluephase G2® low mode) from the same manufacturer, and whether or not other LED-LCU will behave similarly is unknown and needs to be further studied. In conclusion, the findings of this study will help clinicians to be aware of the relative importance of the factors investigated that may lead to overheating and subsequent damage to viable tissue both within the pulp chamber as well as in tissue surrounding the tooth when using LED-LCUs.
